# Off-the-shelf mesenchymal stromal cells derived from umbilical cord tissue

**DOI:** 10.1186/1753-6561-9-S9-P65

**Published:** 2015-12-14

**Authors:** Irene Oliver-Vila, Maria I Coca, Marta Grau-Vorster, Noelia Pujals-Fonts, Marta Caminal, Arnau Pla, Joan García, Joaquim Vives

**Affiliations:** 1 Divisió de Teràpies Avançades/XCELIA, Banc de Sang i Teixits, Edifici Dr. Frederic Duran i Jordà, Passeig Taulat, 116, 08005 Barcelona, Spain

## Background

Human Mesenchymal Stromal Cells (MSC) are multipotent cells residing in all support and structural tissues of the organism and they are capable of extensive expansion in vitro while preserving their identity, potency and genetic stability [1]. Umbilical cord (UC)-derived MSC (UC-MSC) are thought to be more primitive progenitor cells than those isolated from bone marrow or lipoaspirates [2]. The fact that the umbilical cord is a waste tissue is an added value to the isolation of UC-MSC from this source for the generation of cell banks for later use in the allogeneic treatment context as off-the-shelf product [3]. However, several limitations exist regarding the success of the isolation and expansion of UC-MSC up to clinically relevant doses under Good Manufacturing Practice (GMP) environments, provided that current methods 1) require large quantities of tissue processed immediately after birth, 2) are time-consuming, and 3) result in low cellular yields.

In the present study, two methods for UC-MSC derivation from the Wharton's Jelly (WJ) of the UC tissue were tested, namely 1) mechanical scrapping of WJ, and 2) enzymatic digestion of umbilical cord tissue for further use in GMP-compliant cell culture production bioprocesses.

## Materials and methods

**Figure 1:**
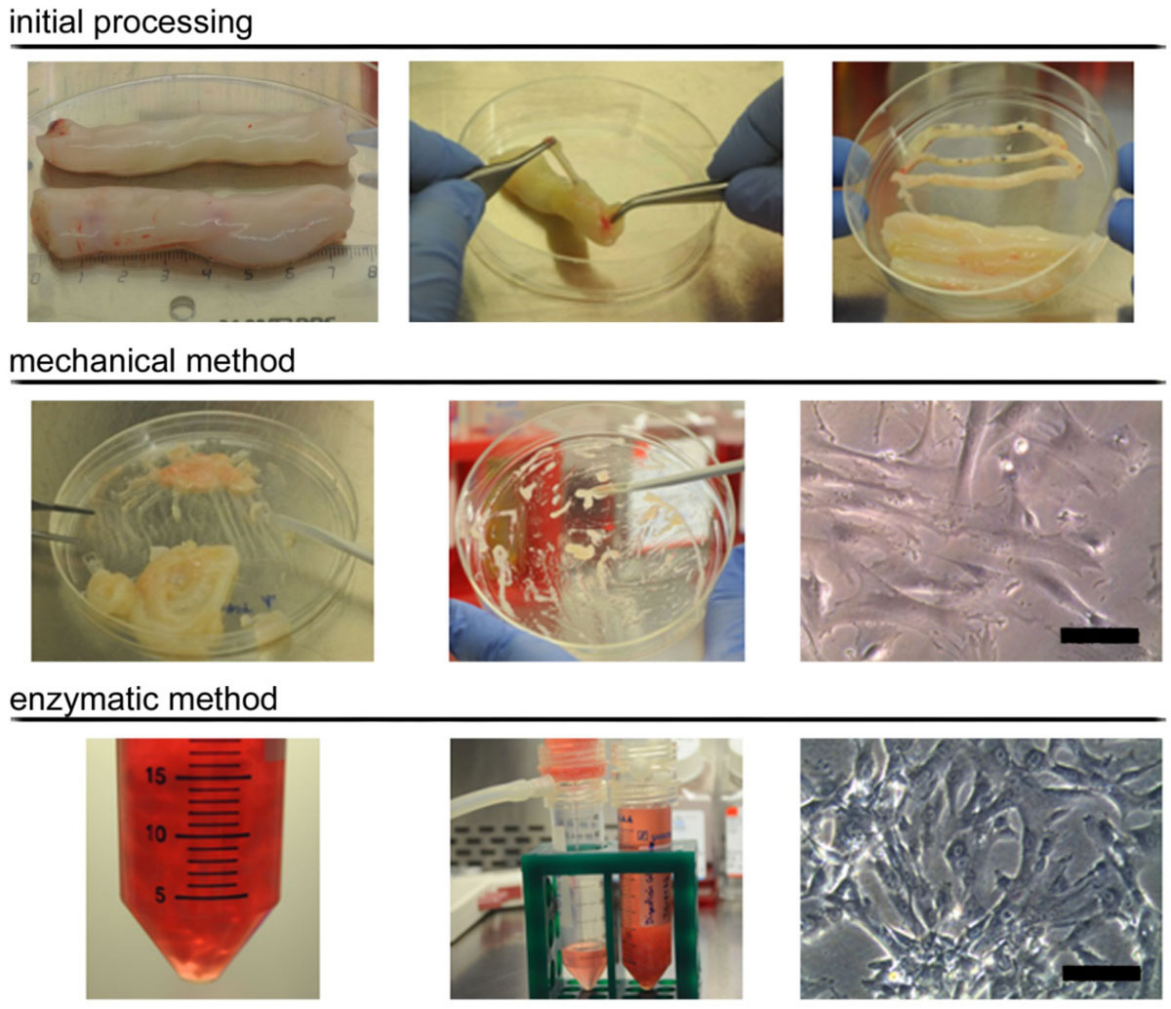
**Derivation of UC-MSC**. Initial processing of the source tissue is shown in the top panel, while the mechanical and enzymatic methods are shown in the middle and bottom panels, respectively. Scale bars= 100 µm (mechanical) & 200 µm (enzymatic).

UC tissue fragments collected in the Concordia Program (http://www.bancsang.net/professionals/en_concordia/) with appropriate donor informed consent were used for sourcing UC-MSC. UC samples were washed twice with sterile Phosphate Buffer Solution (PBS, Gibco) for removing any traces of blood, transferred to a Petri dish and cut into two fragments for testing either one of the two protocols for UC-MSC derivation: mechanical or enzymatic, respectively. In both cases, the fragments were weighted, cut longitudinally and split open to expose the inner surface for then removing the two arteries and the vein (Figure 1, top panel). For the mechanical method, a surgical scalpel was used for scraping the Wharton's Jelly, which was then spread uniformly onto the plastic surface of a 100 mm cell culture plate (Corning) and incubated 30 min at 37ºC (Figure 1, middle panel). In the enzymatic digestion, the tissue was minced and digested with 0.5 mg/mL collagenase I (Sigma-Aldrich) for 2 h and 5 additional minutes with 0.05% trypsin (Gibco) at 37ºC in DMEM (Gibco). Digestion was stopped by adding a final concentration of 5% human Serum B (hSerB, Banc de Sang i Teixits) and passed through 100 µm filters (Millipore) in order to discard undigested tissue fragments (Figure 1, bottom panel). The flow-through was centrifuged at 300g for 10 min. In both cases, subsequent cell expansion was performed using 20 mL of expansion medium consisting of DMEM supplemented with 20% hSerB, 2x104 IU/mL penicillin, 20 mg/mL streptomycin, and 120 µg/mL amphotericin B (Invitrogen).

Cell number and viability were determined by the haemocytometer-based Trypan Blue dye exclusion assay, and the MSC nature of expanded cells was determined according to the criteria established by the International Society for Cellular Therapy [4], including the evaluation of characteristic features such as adherence to plastic and morphology (assessed by bright field microscopy in Leica DMIL LED), immunophenotype by flow cytometry in a FACSCalibur (BD Biosciences). FACS analysis was performed to evaluate expression of surface markers CD31 (555445, BD), CD45 (HI30, BD Biosciences), CD90 (5E10, BD Biosciences), CD73 (AD2, BD Biosciences), CD105 (43A4E1 clone, Miltenyi) and HLA-DR (TU36, BD Biosciences) in a FACS Calibur flow cytometer (BD Biosciences). PE-conjugated IgG1 (X40, BD Biosciences) and FITC-conjugated IgG2bk (27-35, BD Biosciences) antibodies were used as isotype controls. Multipotentiality of UC-MSC was assessed in vitro using StemPro differentiation media (Gibco) and specific stainings for the detection of cell fate into either osteogenic, chondrogenic or adipogenic lineages, were performed as described elsewhere [5].

## Results

Starting umbilical cord tissue was cut into two fragments of same size weighting 7.6 ± 1.9 g and 7.2 ± 1.5 g (n = 10), for testing the mechanical and enzymatic derivation protocols, respectively. Each method presented a series of advantages and disadvantages related to the use of a number of reagents in the case of the enzymatic method and extensive manipulation in an open circuit in the case of the mechanical scrapping method. However, all cases resulted in the successful derivation of at least 1x106 MSC within 24 days, suitable for cryopreservation and subsequent scale up for use in preclinical and/or clinical studies.

Phenotypically, UC-MSC derived following the mechanical method were 99.2% ± 0.7% CD90, 0.6% ± 0.6% CD45, 99.0% ± 0.7%CD73, 0.7% ± 0.8 CD31, 99.1% ± 0.5 CD105, and 0.7% ± 0.8 HLA-DR (n = 3). Similarly, UC-MSC derived following the enzymatic method were 99.1% ± 1.2% CD90, 0.7% ± 1.3% CD45, 97.2% ± 2.5%CD73, 1.5% ± 2.9 CD31, 99.0% ± 1.1 CD105, and 0.7% ± 1.2 HLA-DR (n = 4).

Both methods tested proved to generate cell lines that retained osteogenic, chondrogenic, and adipogenic differentiation potential of the cells as assayed in vitro (Fig. 1). The combination of differentiation assays, growth profiles, morphology assessment and cytometric phenotype confirmed the MSC nature of the cells derived by following the two methodologies described in this study.

## Conclusions

Current methods for MSC derivation from umbilical cord require very fresh tissue within hours from birth, are tedious and yields are low. UC-MSC produced following the methods described in this work are simple, require small quantities of starting material and can be implemented as initial derivation step in any GMP-compliant bioprocess aiming at producing off-the-shelf cryopreserved products for cell therapy.

## Acknowledgements

This project was funded by "La Marató de TV3" (expedient number: 122831), and the Spanish Cell Therapy Network (TerCel; expedient number: RD12/0019/0015).
